# Analysis of effectiveness and safety of cisatracurium infusion during laparoscopic abdominal surgery

**DOI:** 10.3389/fsurg.2025.1686964

**Published:** 2025-12-09

**Authors:** Zhe Hong, Xintong Lin, Weifang Jin

**Affiliations:** 1 Department of Anesthesiology, Shanghai Civil Aviation Hospital, Shanghai, China; 2 Department of Cell & Systems Biology/Department of Human Biology, University of Toronto, Toronto, ON, Canada

**Keywords:** cisatracurium, infusion method, laparoscopic abdominal surgery, neuromuscular monitoring, postoperative recovery

## Abstract

**Objective:**

To explore the efficacy and safety of different infusion modes of cisatracurium in laparoscopic abdominal surgery.

**Methods:**

In this randomized controlled trial, 90 patients undergoing elective laparoscopic abdominal surgery at Shanghai Civil Aviation Hospital (2019–2020) were allocated to either continuous (*n* = 45) or intermittent (*n* = 45) cisatracurium infusion groups. Operation time, dosage of cisatracurium, time of onset, lack of muscle relaxant occurrences, extubation time, recovery index (TOF T1 recovery from 25% to 75%), TOF70% (drug withdrawal to TOF recovery time for 70%), TOF90% (drug withdrawal to TOF recovery time for 90%), mean arterial pressure and heart rate during anesthesia and induction, and occurrence of adverse reactions were compared between the two groups.

**Results:**

While onset time (*P* = 0.102) and operation duration (*P* = 0.946) were comparable between groups, the continuous infusion group demonstrated significant advantages: fewer inadequate relaxation episodes (*P* = 0.003), lower total cisatracurium requirements (*P* < 0.001), and faster recovery (recovery index, TOF70%, and TOF90%; all *P* < 0.001). There was no significant difference in mean arterial pressure and heart rate during the anesthesia and induction between the two groups (*P* = 0.314, *P* = 0.462 and *P* = 0.205, *P* = 0.521). Meanwhile, extubation times (*P* = 0.095) and adverse event rates (*P* = 0.214) showed no significant differences.

**Conclusions:**

Compared with intermittent infusion, continuous infusion of cisatracurium provides better muscle relaxation effect with reduced cisatracurium consumption and faster recovery, without increasing the risk of residual muscle relaxation or adverse reactions in this study, suggesting a favorable safety in laparoscopic abdominal surgery.

## 1 Introduction

The use of muscle relaxants for anesthetic induction in patients undergoing laparoscopic abdominal surgery can effectively reduce coughing triggered by mechanical stimulation, thereby facilitating intraoperative intubation (1). Currently, atracurium and vecuronium are among the muscle relaxants commonly used in clinical anesthesia for laparoscopic abdominal surgery (2). However, atracurium may induce histamine release, potentially triggering adverse events. Conversely, while vecuronium delivered via continuous infusion ensures stable muscle relaxation, it poses a higher risk of residual neuromuscular blockade (3).

Cisatracurium, a stereoisomer of atracurium, is a nondepolarizing skeletal muscle relaxant with an intermediate duration of action (4). It binds to cholinergic receptors at the motor endplate, competitively blocking neuromuscular transmission by antagonizing acetylcholine (5). As an adjunct to general anesthesia, it is used to facilitate endotracheal intubation, provide intraoperative muscle relaxation, or support mechanical ventilation in the ICU, with a lower risk of adverse reactions (6). Clinically, cisatracurium is primarily administered via continuous infusion, intermittent bolus, or target-controlled infusion (TCI) (7). Current research on anesthesia for laparoscopic abdominal surgery, both domestically and internationally, has predominantly focused on other anesthetic agents or techniques, with limited studies investigating the impact of cisatracurium infusion methods (8, 9). To address this gap, our study analyzed 90 patients undergoing elective laparoscopic abdominal surgery at Shanghai Civil Aviation Hospital between January 2019 and January 2020. This study aimed to evaluate the efficacy and safety of different cisatracurium infusion methods in laparoscopic abdominal surgery anesthesia, providing clinical insights to optimize anesthetic outcomes, mitigate the risk of residual neuromuscular blockade, and improve postoperative recovery.

## 2 Materials and methods

### 2.1 General data

**Figure 1:**
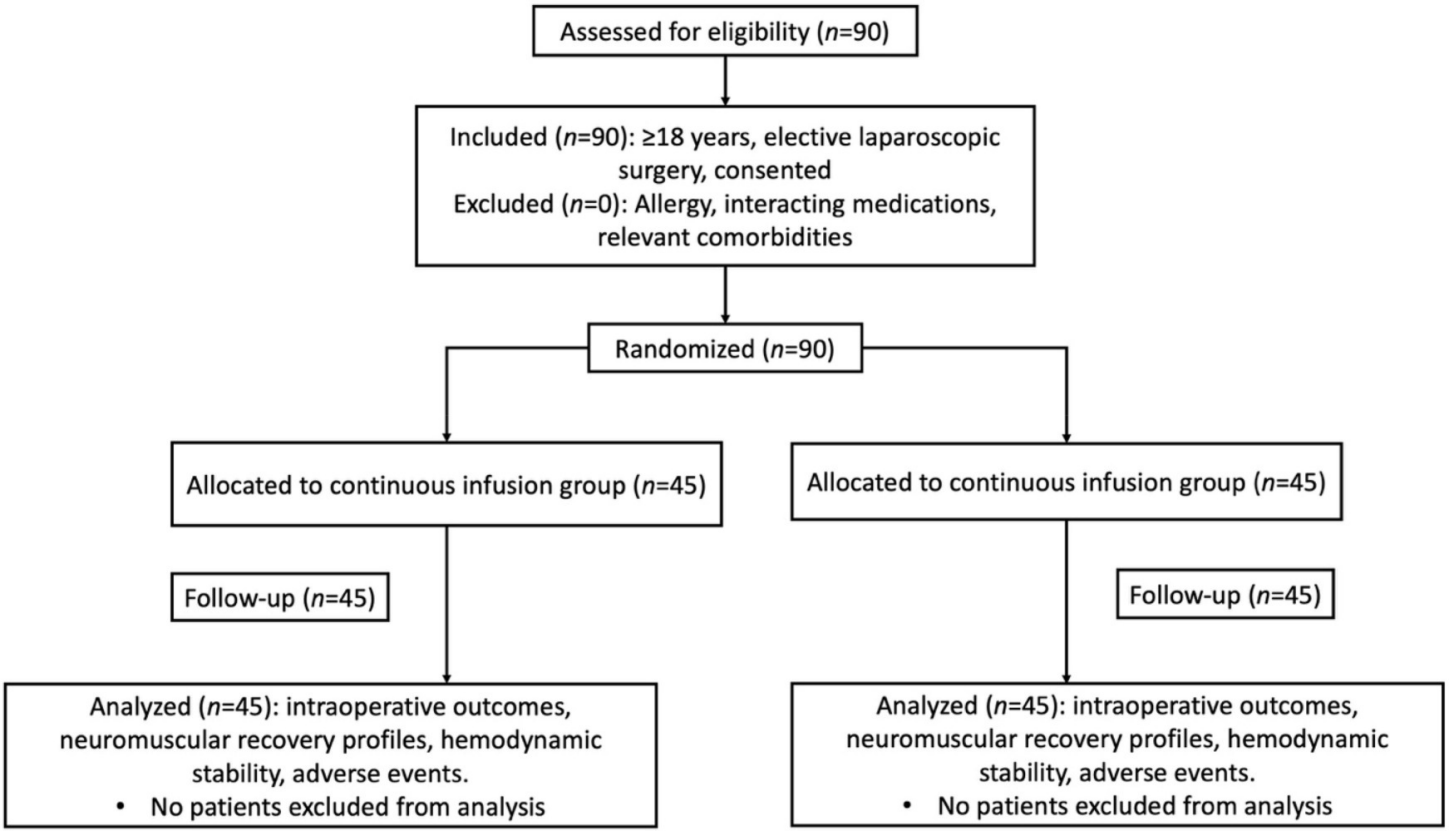
Trial design & flow diagram.

This prospective, randomized controlled trial enrolled 90 patients undergoing elective laparoscopic abdominal surgery under general anesthesia at Shanghai Civil Aviation Hospital between January 2019 and January 2020. Participants included 53 males and 37 females, with a mean age of 51.47 ± 8.36 years. To minimize potential bias, allocation concealment was employed, and outcome assessors were blinded to the group assignments. Using a random number table method, patients were allocated to either the continuous infusion group or the intermittent infusion group (*n* = 45 each). All patients and their families signed informed consent forms after full disclosure of the study protocol (Figure 1).

Inclusion criteria were: (1) Age ≥18 years; (2) Scheduled for elective laparoscopic abdominal surgery under general anesthesia; (3) Willing and able to comply with study procedures.

Exclusion criteria were: (1) Known allergy or contraindication to cisatracurium; (2) Use of medications affecting neuromuscular transmission within 1 month preoperatively; (3) Neuromuscular disorders; (4) Cognitive impairment or psychiatric diseases.

### 2.2 Anesthesia protocol

All patients underwent comprehensive preoperative evaluations to exclude surgical contraindications and observed an 8 h fast (no food or water). Upon patient arrival in the operating room, vital signs were monitored, and intravenous access was obtained. Neuromuscular function was monitored using a neuromuscular transmission monitor (TOF-Watch SX, Beijing Zhongxi Yuanda Technology Co., Ltd.).

Anesthesia was induced with: intravenous injection of 0.06 mg/kg midazolam [Jiangsu Nhwa Pharmaceutical Co., Ltd., National Medical Products Administration (NMPA) Approval No. H20031037], 0.4 μg/kg sufentanil (Yichang Humanwell Pharmaceutical Co., Ltd., NMPA Approval No. H200541720), and 2 mg/kg propofol (Jiangsu Nhwa Pharmaceutical Co., Ltd., NMPA Approval No. H20123137).

After the patient lost consciousness, the TOF-Watch SX was calibrated to maintain a baseline train-of-four response of 100% ± 10%. Five minutes later, 0.15 mg/kg cisatracurium (Jiangsu Hengrui Pharmaceuticals Co., Ltd., NMPA Approval No. H20060869) was administered intravenously. Endotracheal intubation was performed when T1 of TOF stimulation reached 0, followed by mechanical ventilation.

Anesthesia was maintained with target-controlled infusion (TCI) of propofol, with TOF monitoring continued throughout surgery. The post-tetanic count (PTC) was monitored when T1 = 0, and episodes of inadequate relaxation (PTC ≥3) were recorded and managed according to the group assignment. For the continuous infusion group, an initial cisatracurium infusion of 1.5 μg/(kg·min) was started upon PTC ≥3, with the rate increased by 10% for any recurrence to maintain a PTC ≤2. For the intermittent infusion group, a 0.1 mg/kg cisatracurium bolus was administered intermittently for each PTC ≥3 event.

Muscle relaxants were discontinued 10 min before surgery ended. At T1 recovery to 25% of baseline, neostigmine was administered for reversal.

### 2.3 Observation indicators

#### 2.3.1 Intraoperative outcomes

The following intraoperative variables were compared between groups: surgical duration, total cisatracurium consumption, the onset time of neuromuscular blockade, and the frequency of inadequate relaxation, the latter being defined as PTC ≥3.

#### 2.3.2 Neuromuscular recovery profiles

Recovery parameters, including the time to tracheal extubation, the recovery index (T1 recovery from 25% to 75% of baseline TOF ratio), and the time to achieve a TOF ratio recovery of 70% (TOF70%) and 90% (TOF90%) following the discontinuation of the muscle relaxant, were assessed for both groups.

#### 2.3.3 Hemodynamic stability

Mean arterial pressure (MAP) and heart rate (HR) were recorded and compared during anesthesia induction and maintenance phases.

#### 2.3.4 Adverse events

The incidence of adverse reactions was documented, including facial flushing, difficulty opening eyes, post-extubation weakness, bronchospasm, and other anesthesia-related complications.

Intraoperative outcomes were compared between the two groups: Surgical duration, total cisatracurium consumption, onset time, and frequency of inadequate muscle relaxation (PTC ≥3) were recorded for both groups.

(2) Neuromuscular recovery profiles were compared between the two groups: Time to tracheal extubation, recovery index (time for T1 to recover from 25% to 75% of baseline TOF ratio), TOF70% (time from muscle relaxant discontinuation to TOF ratio recovery of 70%), and TOF90% (time from discontinuation to TOF ratio recovery of 90%) were recorded for both groups.

(3) Mean arterial pressure (MAP) and heart rate (HR) during anesthesia maintenance and induction phases were compared between the two groups.

(4) Adverse events were compared between the two groups: Incidence of facial flushing, difficulty opening eyes, post-extubation weakness, bronchospasm, and other adverse reactions were documented.

### 2.4 Statistical analysis

A *post-hoc* analysis confirmed that the cohort of 90 patients was generally adequate for a single center randomized study. Data were processed using SPSS 22.0. Normally distributed continuous variables were expressed as mean ± standard deviation (mean ± *s*) and analyzed with Student's *t*-test. Count data were presented as number of cases (*n*), percentages (%), or median (interquartile range) and compared using *χ*^2^ tests. A two-tailed *P* < 0.05 was considered statistically significant.

## 3 Results

### 3.1 Baseline characteristics

No statistically significant differences (all *P* > 0.05) were observed between groups in gender, age, body mass index (BMI), or American Society of Anesthesiologists (ASA) physical status classification, confirming comparability of the groups (Table 1).

**Table 1:** Comparison of baseline characteristics between groups.

Index		Continuous infusion (*n* = 45)	Intermittent infusion (*n* = 45)	*t*/*χ*^2^ value	*P*-value
Gender	Male	27	26	*χ*^2^ = 3.568	0.054
	Female	18	19		
Mean age (years)		51.47 ± 8.37	41.46 ± 8.38	*t* = 1.252	0.092
BMI (kg/m^2^)		22.84 ± 2.36	22.34 ± 2.64	*t* = 0.874	0.312
ASA classification	II	30	29	*χ*^2^ = 1.953	0.076
	III	15	16		

### 3.2 Intraoperative outcomes

No significant differences were observed between the two groups in cisatracurium onset time (*P* = 0.102) or surgical duration (*P* = 0.946). However, the continuous infusion group demonstrated significantly fewer inadequate muscle relaxation episodes (*P* = 0.003) and lower total cisatracurium consumption (*P* < 0.001) (Table 2).

**Table 2:** Intraoperative comparisons between groups.

Index	Continuous infusion (*n* = 45)	Intermittent infusion (*n* = 45)	*t*/*χ*^2^ value	*P* value
Surgical duration (min)	158.67 ± 39.12	172.83 ± 43.51	*t* = 1.623	0.946
Cisatracurium dose (mg)	37.32 ± 4.63	45.15 ± 5.14	*t* = 14.824	<0.001
Onset time (min)	3.39 ± 0.56	3.51 ± 0.58	*t* = 1.234	0.102
Inadequate muscle relaxation episodes	5 (2–6)	8 (4–9)	*χ*^2^ = 5.385	0.003

### 3.3 Neuromuscular recovery outcomes

No significant difference was found in extubation time between the two groups (*P* = 0.095). The continuous infusion group demonstrated significantly shorter recovery index (*P* < 0.001), TOF70% (*P* < 0.001), and TOF90% (*P* < 0.001) (Table 3).

**Table 3:** Comparison of neuromuscular recovery profiles between groups (mean ± s).

Index	Continuous infusion (*n* = 45)	Intermittent infusion (*n* = 45)	*t* value	*P* value
Extubation time (min)	54.81 ± 11.61	54.68 ± 11.63	1.325	0.095
Recovery index (min)	14.56 ± 2.14	19.35 ± 2.61	10.314	<0.001
TOF70% (min)	47.23 ± 10.82	52.31 ± 8.53	11.237	<0.001
TOF90% (min)	28.31 ± 3.56	35.24 ± 3.76	10.328	<0.001

### 3.4 Comparison of mean arterial pressure and heart rate during anesthesia maintenance and induction periods

No significant intergroup differences were observed in mean arterial pressure (MAP) or heart rate (HR) during anesthesia maintenance or induction phases (all *P* > 0.05) (Table 4).

**Table 4:** Comparison of MAP and HR during anesthesia maintenance and induction periods between groups (mean ± s).

Index	Continuous infusion group (*n* = 45)	Intermittent infusion group (*n* = 45)	*t* value	*P* value
Mean arterial pressure (mmHg)				
Maintenance period	92.16 ± 4.72	92.15 ± 4.81	1.021	0.314
Induction period	78.92 ± 4.15^a^	78.89 ± 4.18 ^a)^	1.231	0.205
Heart rate (beats/min)				
Maintenance period	78.95 ± 11.02	79.05 ± 10.96	0.952	0.462
Induction period	78.63 ± 10.95	78.86 ± 11.01	0.854	0.521

a Compared with maintenance period, *P* < 0.05.

### 3.5 Comparison of adverse reaction incidence between groups

The overall incidence of adverse reactions showed no statistical difference (*P* = 0.214) (Table 5).

**Table 5:** Comparison of adverse reaction incidence between groups.

Index	Continuous infusion group (*n* = 45)	Intermittent infusion group (*n* = 45)	*χ*^2^ value	*P* value
Facial flushing	1	0	–	–
Difficulty opening eyes	1	1	–	–
Post-extubation weakness	0	1	–	–
Bronchospasm	0	0	–	–
Total incidence (%)	2 (4.44)	2 (4.44)	1.125	0.214

## 4 Discussion

Muscle relaxants, also called skeletal muscle relaxants or N2 cholinergic receptor blockers, selectively act on N2 receptors at motor endplate membranes (10), blocking neuromuscular transmission and causing muscle relaxation. Based on their mechanism of action, they can be divided into depolarizing muscle relaxants and non-depolarizing muscle relaxants (11). Their use has changed the practice of relying solely on deepening anesthesia for muscle relaxation, making them important adjuncts in general anesthesia surgery (12). Their application not only enables rapid endotracheal intubation but also expands the surgical field, facilitating precise operations in thoracic or abdominal cavities (13). However, muscle relaxants may cause significant hemodynamic changes by stimulating or inhibiting peripheral autonomic nerves, histamine release, and production of vasoactive substances, leading to adverse reactions. Residual muscle relaxation may cause postoperative pulmonary dysfunction, airway obstruction and other complications, seriously affecting patients’ quality of life (14).

Cisatracurium, an intermediate-acting non-depolarizing muscle relaxant, offers distinct pharmacological advantages. Unlike other agents in its class, it demonstrates minimal effects on cardiovascular parameters and negligible histamine release (15). The impact on liver and kidney function is minimal, and no dose adjustment is required for elderly patients or those with renal or hepatic impairment (15). Previous studies have suggested that cisatracurium infusion methods may influence postoperative residual muscle relaxation (16). Therefore, investigating the efficacy and safety of different cisatracurium infusion methods in laparoscopic abdominal surgery anesthesia has important clinical significance for improving anesthetic effects, reducing risks of residual muscle relaxation, and enhancing patients’ quality of life.

Our study compared two administration protocols: continuous infusion (1.5 μg/kg/min) and intermittent boluses (0.1 mg/kg). The comparability of baseline characteristics, including age, BMI, and ASA classification, between the two groups (Table 1) enhances the internal validity of our study, minimizing the likelihood of confounding and reinforcing confidence that the observed differences are attributable to the infusion method. Continuous cisatracurium infusion is calculated based on patient weight and administered at 1.5 μg/(kg·min) until T1 recovers to 25% of baseline, when neostigmine is given for reversal (17). Intermittent intravenous cisatracurium administration involves giving additional 0.1 mg/kg intravenous boluses at 25–30 min intervals, with dosing adjusted according to the patient's clinical muscle relaxation status (18). Previous work by Li et al. found that continuous cisatracurium infusion for maintaining deep muscle relaxation in laparoscopic abdominal surgery was safe and effective with high satisfaction (19). Although recovery time after discontinuation was slightly longer, there was no significant effect on postoperative residual muscle relaxation (19). Meanwhile, the continuous infusion group had significantly higher average muscle relaxant usage and surgeon satisfaction scores at 0, 1, and 2 h after surgery began compared to the intermittent infusion group (19). Ran et al. found that continuous intravenous cisatracurium reduced drug consumption in elderly patients undergoing laparoscopic gastrointestinal tumor surgery, facilitating postoperative muscle relaxation recovery (20). Ran et al. also reported significantly shorter time to TOF70% recovery, lower recovery index, total drug consumption and average infusion rate compared to intermittent infusion (20). Our results showed that the continuous cisatracurium infusion group had significantly fewer occurrences of inadequate muscle relaxation than the intermittent infusion group, indicating that continuous infusion of cisatracurium provides better muscle relaxation effects in laparoscopic abdominal surgery anesthesia. This is consistent with previous findings by Li et al. However, regarding cumulative cisatracurium consumption, our results differed from previous studies. This discrepancy is likely because our continuous infusion rate of 1.5 μg/(kg·min) was lower than the 0.2 mg/(kg·h) dose used in Li et al.'s study. Consequently, in our study, continuous infusion reduced cisatracurium consumption compared with intermittent infusion in laparoscopic abdominal surgery anesthesia. The recovery index, TOF70%, and TOF90% were significantly shorter in the continuous infusion group, indicating faster recovery of muscle relaxation, which is also consistent with Ran et al.'s conclusions. Importantly, this accelerated pharmacologic recovery did not result in a statistically significant difference in the clinical endpoint of time to tracheal extubation. The minimal difference observed suggests that, in practice, both infusion methods are equivalent for achieving timely extubation, which is a key consideration for anesthesiologists.

Hu et al. found no significant differences in adverse reactions like nausea and dizziness between closed-loop target-controlled cisatracurium infusion and intermittent infusion (21). Zhang et al. found no statistical differences in mean arterial pressure or heart rate between different administration methods (22). Furthermore, patients receiving either continuous or intermittent infusion at different doses showed no adverse reactions such as regurgitation or aspiration (22). Our results showed no significant differences in mean arterial pressure or heart rate during anesthesia maintenance or induction between groups, nor in adverse reaction rates in this cohort, suggesting that different infusion methods may have relatively small effects on patients’ blood pressure and heart rate. Within the limits of this study, neither infusion method appeared to increase the risk of adverse reactions, demonstrating a favorable safety profile. These findings are consistent with the safety profiles reported by Hu et al. and Zhang et al, though larger studies are needed to definitively compare adverse event rates.

This study has several limitations that should be considered. Firstly, it was conducted at a single center with a relatively limited sample size. While our *post-hoc* analysis indicated sufficient power for the primary efficacy outcomes, the single-center design may affect the generalizability of our findings. The patient population, surgical teams, and anesthetic protocols at our institution may not be fully representative of those in other settings. Therefore, future multicenter studies with larger cohorts would be valuable to confirm and extend our findings across diverse clinical environments.

In conclusion, compared with intermittent infusion, continuous cisatracurium infusion in laparoscopic abdominal surgery provides better muscle relaxation, reduces cisatracurium consumption, and shortens muscle relaxation recovery time. Additionally, no increase in postoperative neuromuscular blockade or adverse reactions was observed in this study. These results suggest a favorable safety profile for continuous cisatracurium infusion in this context, but its assessment was limited by the low number of adverse events.

## Data Availability

The original contributions presented in the study are included in the article/Supplementary Material, further inquiries can be directed to the corresponding author.
